# Antibiofilm and antivirulence activities of laminarin-gold nanoparticles in standard and host-mimicking media

**DOI:** 10.1007/s00253-024-13050-4

**Published:** 2024-02-13

**Authors:** Nazia Tabassum, Fazlurrahman Khan, Geum-Jae Jeong, Dokyung Oh, Young-Mog Kim

**Affiliations:** 1 Marine Integrated Biomedical Technology Center, The National Key Research Institutes in Universities, Pukyong National University, Busan, 48513 Republic of Korea; 2 Research Center for Marine Integrated Bionics Technology, Pukyong National University, Busan, 48513 Republic of Korea; 3 Institute of Fisheries Sciences, Pukyong National University, Busan, 48513 Republic of Korea; 4 Department of Food Science and Technology, Pukyong National University, Busan, 48513 Republic of Korea

**Keywords:** Antibiofilm, Laminarin, Gold nanoparticles, Antivirulence, *Pseudomonas aeruginosa*, *Staphylococcus aureus*, Host-mimicking media

## Abstract

**Abstract:**

The rapidly rising antimicrobial resistance (AMR) in pathogenic bacteria has become one of the most serious public health challenges, with a high death rate. Most pathogenic bacteria have been recognized as a source of AMR and a primary barrier to antimicrobial treatment failure due to the development of biofilms and the production of virulence factors. In this work, nanotechnology was employed as a substitute method to control the formation of biofilms and attenuate virulence features in *Pseudomonas aeruginosa* and *Staphylococcus aureus*. We synthesized biocompatible gold nanoparticles from marine-derived laminarin as potential biofilm and virulence treatments. Laminarin-gold nanoparticles (Lam-AuNPs) have been identified as spherical, 49.84 ± 7.32 nm in size and − 26.49 ± 1.29 mV zeta potential. The MIC value of Lam-AuNPs against several drug-resistant microbial pathogens varied from 2 to 1024 μg/mL in both standard and host-mimicking media. Sub-MIC values of Lam-AuNPs were reported to effectively reduce the production of *P. aeruginosa* and *S. aureus* biofilms in both standard and host-mimicking growth media. Furthermore, the sub-MIC of Lam-AuNPs strongly reduced hemolysis, pyocyanin, pyoverdine, protease, and several forms of flagellar and pili-mediated motility in *P. aeruginosa*. Lam-AuNPs also inhibited *S. aureus* hemolysis and the production of amyloid fibrils. The Lam-AuNPs strongly dispersed the preformed mature biofilm of these pathogens in a dose-dependent manner. The Lam-AuNPs would be considered an alternative antibiofilm and antivirulence agent to control *P. aeruginosa* and *S. aureus* infections.

**Key points:**

*• Lam-AuNPs were biosynthesized to control biofilm and virulence.*

*• Lam-AuNPs show effective biofilm inhibition in standard and host-mimicking media.*

*• Lam-AuNPs suppress various virulence factors of P. aeruginosa and S. aureus.*

**Supplementary Information:**

The online version contains supplementary material available at 10.1007/s00253-024-13050-4.

## Introduction

The entire world is dealing with infections caused by a diverse spectrum of bacterial pathogens, with an increasing frequency of death (Ikuta et al. 2022). The development of antimicrobial resistance (AMR) characteristics by pathogenic bacteria is the leading cause of antimicrobial treatment failure (Murray et al. 2022). Several resistance mechanisms in bacterial pathogens have been investigated, and additional mechanisms are continually being examined (Reygaert 2018). The development of biofilm has been recognized as one of the adaptive resistance mechanisms and has become a prominent cause of antimicrobial failure (Coenye et al. 2022; Mah and O’Toole 2001). The biofilm is an assembly of bacterial cells covered in self-produced extracellular polymeric biomolecules such as DNA, polysaccharides, and proteins (Karygianni et al. 2020). Biofilm’s strong adhesive qualities make it very susceptible to attachment to both abiotic (e.g., medical equipment) and biotic (e.g., skin, teeth, and epithelial cell surface of the trachea and urinary system interfaces) surfaces (Dunne 2002; Khatoon et al. 2018). The biofilm also acts as a safety reservoir for mutant and persister microbial cells, which are resistant and tolerant to antimicrobial treatments and responsible for persistent infections (Coenye et al. 2022; Khan et al. 2020). WHO has identified biofilm-forming bacterial pathogens such as *Staphylococcus aureus*, *Acinetobacter baumannii*, *Pseudomonas aeruginosa*, *Enterococcus faecium*, *Klebsiella pneumoniae*, and *Enterobacter* spp. as priority pathogens against which drugs must be discovered (De Oliveira et al. 2020; Rice 2008).

In addition to biofilm production, these pathogens generate a wide range of virulence factors that assist in adhesion, invasion of host cells, and evasion of the host’s immune system (Qin et al. 2022). Disarming bacterial virulence is another possible strategy for bacterial infection control (Rasko and Sperandio 2010). As a result, there is always a significant need to develop drugs that can inhibit the biofilm and decrease the virulence qualities of bacteria. Furthermore, the majority of antimicrobial screens were conducted against planktonic cells using a standard growth medium, which may not be effective in the host’s physiological environment (Ersoy et al. 2017; Garcia Maset et al. 2022; Humphries et al. 2018; Kirchner et al. 2012; Kubicek-Sutherland et al. 2015). As a result, it is important to consider physiological conditions while assessing antibiotic activity and attenuating biofilm formation and virulence features (Ersoy et al. 2017; Kang et al. 2023, 2022; Richter et al. 2020).

Due to various benefits, green-synthesized nanoparticles (NPs) have recently gained popularity in treating microbial infections (Khan et al. 2022). The number of studies on the green synthesis of NPs for antibacterial activity is increasing, although the majority of them use crude extracts derived from plants, algae, animals, and microorganisms (Javaid et al. 2018; Jeong et al. 2022; Naidi et al. 2021). Aside from extracts, attention should be given to synthesizing NPs using purely natural substances in order to replicate the synthesis with the same physicochemical properties (Khan et al. 2022, 2021). The marine organism serves as a repository for the bioactive material produced, which may be used in various biological activities (Ghosh et al. 2022; Jeong et al. 2022; Wan et al. 2021). This study aimed to synthesize gold nanoparticles (AuNPs) utilizing the pure marine-derived compound laminarin (Lam). Lam is a polysaccharide derived from brown algae (e.g., *Laminaria*, *Saccharina*, and *Eisenia* spp.) that has been widely used in therapeutics as an anticoagulant, antioxidant, anticancer, and anti-inflammatory drug (Zargarzadeh et al. 2020). In addition, Lam has been found to be a source of dietary fiber as well as an intestinal modulator (Devillé et al. 2004; Rattigan et al. 2020). The antimicrobial activity of the produced laminarin-gold nanoparticles (Lam-AuNPs) against several drug-resistant microbial pathogens was tested in standard and host-mimicking media such as synthetic human urine (SHU), artificial saliva, and artificial sputum. A comprehensive investigation of the inhibitory effects of Lam-AuNPs on biofilm and the attenuation of several virulence features of *P. aeruginosa* and *S. aureus* was also conducted.

## Materials and methods

### Reagents, microbial pathogens, and experimental conditions

Gold(III) chloride trihydrate and Lam (CAS number 9008–22-4), mucin from the porcine stomach (CAS number 84082–64-4), diethylene triamine penta-acetic acid (DTPA), salmon sperm DNA, and Congo red were received from Sigma-Aldrich Co. (St. Louis, MO, USA). The casamino acid and egg yolk emulsion (Cat. No. MB-E1864) were purchased from MBcell (Kisan Bio Co., Ltd., South Korea). *S. aureus* (KCTC1916), *P. aeruginosa* PAO1 (KCTC1637), *Listeria monocytogenes* (KCTC3569), and *Escherichia coli* (KCTC1682) were bought from the Korean Collection for Type Cultures (KCTC, Daejeon, South Korea). The Korean Culture Center of Microorganisms (KCCM; Seodaemun-gu, Seoul) provided the *Streptococcus mutans* (KCCM40105) and *Candida albicans* (KCCM11282), while the American Type Culture Collection supplied the *K. pneumoniae* (ATCC4352). All the bacterial cells were cultured in tryptic soy broth (TSB), whereas *C. albicans* was cultivated in potato dextrose broth (PDB). The growth temperature for these microbes was 37 °C.

### Artificial host-mimicking media

#### Artificial sputum media

As discussed earlier, the composition and procedure for making artificial sputum media were followed (Sriramulu et al. 2005). Briefly, the procedure for the preparation of 1 L sputum media includes the following steps: We have sequentially added 5 g mucin, 4 g DNA, 5.9 g DTPA, 5 g NaCl and 2.2 KCl, and 1.81 g tris base in water followed by continuous stirring. The pH of the media was adjusted to 7.0 using tris base. After autoclaving at 121 °C for 15 min, the egg yolk emulsion with a volume of 5 mL was added to the media. The filter-sterilized amino acid (250 mg) was added to the media.

#### Artificial human saliva

The artificial human saliva, which is available commercially, was obtained from Sigma-Aldrich Co. (SAE0149-200ML).

#### SHU

The preparation of the SHU was followed as described earlier by Brooks and Keevil (1997). The pH of the SHU was adjusted to 6.5 to avoid forming the precipitate.

### Green synthesis of Lam-AuNP

The green synthesis of the AuNPs was carried out in the same manner as stated previously, with only minor modifications (Kang et al. 2023). Briefly, the alkaline (pH 9.0) solution of HAuCl_4_·3H_2_O (1 mM) was dissolved in the deionized water (volume of 200 mL) and stirred at 60 °C. At the same time, the solution of Lam (0.2%) made in water was added to the gold(III) chloride trihydrate solution, and the mixture was allowed to be stirred constantly. The yellow color of the initial reaction mixture was continuously changed to red wine, which was employed as a preliminary indicator for forming AuNPs. The appearance of an absorption spectrum was monitored in situ by scanning spectra (from 200 to 900 nm). The liquid Lam-AuNPs solution was freeze-dried into powder form.

### Characterization of Lam-AuNPs

Different other instruments such as Fourier transform infrared spectrometer [FTIR; JASCO (FT-4100), Tokyo, Japan], field emission transmission electron microscopy (FE-TEM; JEM-F200, JEOL, Japan), and X-ray diffractometer [XRD; X-Ray Diffractometer, Rigaku (Japan), Ultima IV] were used to determine the surface chemistry, morphology, and crystalline nature of Lam-AuNPs. A particle analyzer (Litesizer 500; Anton Paar, GmbH) was used to measure Lam-AuNP size and zeta potential. The same FE-TEM apparatus was used to analyze Lam-AuNPs energy dispersive spectroscopy (EDS).

### Estimation of minimum inhibitory concentration (MIC) of Lam-AuNPs

The MIC value of Lam-AuNPs was evaluated using the same instruction given by the Clinical and Laboratory Standards Institute 2016 (Wayne 2011). The cell culture of each microbe [optical density (OD)_600_ = 0.05] prepared in TSB/PDB was filled in the microtiter plate containing Lam-AuNPs (from 16 to 2048 μg/mL). The plate was incubated at 37 °C for 24 h. The OD_600_ of the cell culture was determined. In addition, the visible cell growth was also checked. The MIC value was determined based on more than 90% growth inhibition in the presence of NPs, as previously explained (Konaté et al. 2012). The colony-counting approach was used to ascertain the MIC of Lam-AuNPs against drug-resistant microbial pathogens in the host-mimicking media (Tabassum et al. 2023a). The microbial cell culture (100 μL) was diluted up to 10^−8^ dilutions in each well that contained different concentrations of Lam-AuNPs (from 16 to 2048 μg/mL). The cell culture (100 μL) was spread-plated for bacterial cells on TSA plates and for *C. albicans* cells on PDA plates. The microbial colonies that appeared on the agar plates were counted. The MIC values were determined at a given concentration when there was a cell reduction of more than 90%. The experiments were run in triplicate.

### Biofilm assays in the standard and host-mimicking media

Biofilm formation of *P. aeruginosa* and *S. aureus* was carried out in standard media supporting microbial growth, such as TSB media and several types of host-mimicking growth media. The host-mimicking media used in this study include artificial saliva media, SHU, and artificial sputum media. The previously described procedures were used to conduct the tests for the biofilm of these bacteria both in the presence of Lam-AuNPs and in the absence of Lam-AuNPs (Khan et al. 2021). The assessment of the biofilm inhibition was made using both crystal violet staining and colony-counting approaches. The cell culture (OD_600_ = 0.05) prepared in all three media was incubated in a 96-well microtiter plate that contained Lam-AuNPs (16 to 512 μg/mL) and further incubated at 37 °C for 24 h. Crystal violet dye was used to stain the surface-attached microbial biofilm cells. The clean-dried-stained plates were eventually filled with 95% ethyl alcohol to dissolve the biofilm cells, and the OD at 570 nm was measured.

In order to test the inhibitory effect of Lam-AuNPs against microbial pathogens in host-mimicking conditions, the colony-counting method was adopted (Tabassum et al. 2023a). Briefly, the cell culture prepared in host-mimicking media such as SHU, saliva, and sputum was added to the microplate containing a range of Lam-AuNPs (16 to 2048 μg/mL). The microplate was kept in an incubator at 37 °C for 24 h. The colonies of planktonic and biofilm cells from each set of experiments in different host-mimicking media were enumerated as described earlier (Tabassum et al. 2023a). Planktonic cell culture (100 μL) was serially diluted up to 10^−8^, whereas the biofilm cells, after three times washing, were scraped off and resuspended in TSB media. Similar to planktonic cell culture, biofilm cell culture was also serially diluted. The diluted planktonic and biofilm cells in TSB media were spread-plated on tryptic soy agar (TSA) plates. Based on the counted colonies on the TSA plates, the colony-forming unit (CFU) value from the test samples was compared with the control samples.

### Dispersal effect of Lam-AuNPs towards mature biofilm

The disinfection of mature biofilms of bacterial pathogens by Lam-AuNPs was carried out as performed earlier (Kang et al. 2023). A mature biofilm (24 h old) was established in a 96-well microtiter plate by incubating the bacterial cell culture (OD_600_ = 0.05). After removing the unattached (i.e., planktonic) cells, the adhered cells were washed using TSB. Different concentrations of Lam-AuNPs ranging from 64 to 2048 μg/mL prepared in TSB were transferred to each well containing washed adhered biofilm cells. Further incubation in the same conditions (24 h at 37 °C) was carried out. Using the same method used for the biofilm inhibition experiment, the effect of Lam-AuNPs on the mature biofilm was determined by staining it with crystal violet. The experiments were run in triplicate.

### Imaging of the biofilm architecture

Scanning electron microscopy (SEM) was used to examine the biofilm cells that were treated and untreated with Lam-AuNPs (Kang et al. 2023). The establishment of biofilm was carried out on the nylon membrane (0.5 × 0.5 cm). The cell culture (OD_600_ = 0.05) was placed in the 24-well microplate containing a nylon membrane for each. The sub-MIC concentration (128 μg/mL) of Lam-AuNPs was also added to these wells. The untreated cell was considered as a control group. The biofilm cells on the membrane surface were directly fixed using 2% formaldehyde and 2.5% glutaraldehyde. After removing the unattached (i.e., planktonic) cells, biofilm cells were washed using phosphate-buffered saline (PBS) (pH 7.4) and dehydrated using different ethanol concentrations. After freeze-drying, the biofilm cells were imaged using the TESCAN Vega II LSU microscope (Tescan, Brno, Czech Republic).

### Antivirulence properties of Lam-AuNPs

The virulence attenuating effect of Lam-AuNPs towards several types of virulence properties of *S. aureus* and *P. aeruginosa* was carried out, as discussed earlier (Kang et al. 2022). The swarming, swimming, and twitching motility in *P. aeruginosa* were carried out using different agar media types. The agar media prepared in Luria–Bertani (LB) for swarming contained 0.4% casamino acid, 0.5% glucose, and 0.5% bacto agar. The agar media for swimming contained 0.25% NaCl, 1% tryptone, and 0.3% bacto agar. Meanwhile, the twitching media prepared in LB contained 30 mM glucose, 0.2% casamino acid, and 1.5% bacto agar. *P. aeruginosa* cell culture (3 μL) was put in the middle of swarming and swimming agar plates. However, in the case of twitching, the agar medium was poured into the plate, which was deposited with cell culture in the center. In the swarming and swimming motility experiment, the diameter of the traveled cells was measured after incubating them for 24 h at 37 °C. However, the agar medium was removed in the twitching, and the surface traveled cells were dyed with crystal violet. After that, the diameter of the cells was measured.

The study of the impact of Lam-AuNPs on the production of virulence factors (e.g., pyocyanin, pyoverdine, protease, and hemolytic factor) by *P. aeruginosa* was carried out. In each case, the concentration of Lam-AuNPs was 16 to 128 μg/mL. The pyocyanin production was estimated using the colorimetric method and extracted using chloroform and 0.2 N HCl. The pink color pyocyanin pigment was quantified at OD_520_. The siderophore (e.g., pyoverdine) production in the presence of Lam-AuNPs was carried out by growing cells in a minimal salt medium containing 2% sodium succinate (Khan et al. 2021). The green pigment pyoverdine was quantified at OD_405_. A skim-milk agar plate was utilized to determine the impact of Lam-AuNPs on the protease activity of *P. aeruginosa*. The Lam-AuNPs treated and untreated cell cultures were subjected to centrifugation, and the resulting supernatant underwent filter sterilization utilizing a 0.2 μm filter. The supernatant (30 μL) was pipetted into the hole prepared in the skim-milk agar plate. The protease-digested protein zone was measured. The effect on the hemolytic activity of Lam-AuNPs towards both *S. aureus* and *P. aeruginosa* was carried out by mixing diluted sheep blood with Lam-AuNPs treated and untreated cell cultures. The antihemolytic effect was determined by measuring the OD_543_. To check the amyloid fibril formation from *S. aureus*, the Congo-red staining approach was employed to visualize the antivirulence effect of Lam-AuNPs. The visualization of amyloid fiber formation was checked on a TSA plate containing Congo red (40 µg/mL). These plates were also added with different concentrations of Lam-AuNPs. The cell culture that grew overnight was streaked on the agar plate and then incubated at 37 °C for 24 h. The appearance of the red, rough, and dry colony on the surface of the Congo-TSA plate indicates the production of the amyloid fiber from *S. aureus*.

### Cell cytotoxicity assays

The mouse-derived macrophage RAW 264.7 cell line was used to test the cell cytotoxicity of Lam-AuNPs, as previously reported (Khan et al. 2021). Briefly, the harvested RAW 264.7 cell culture was distributed in a 96-well microtiter plate and incubated for 48 h at 37 °C. Following incubation, the cell was exposed to several doses of Lam-AuNPs (1 to 1024 μg/mL) and kept at 37 °C for 24 h. The supernatant from the culture was thrown away, the cells were rinsed with PBS, and then a new culture medium was added to each well. The MTT [(3,4,5-dimethylthiazol-2-yl)-2–5-diphenyl tetrazolium bromide] dye, which was prepared in PBS, was added to each well, and then the mixture was incubated at 37 °C for 3 h. The entire media was discarded, added with DMSO in each well, and further incubated for 3 h at 37 °C. The fluorescence of the dye was quantified at a wavelength of 570 nm. The investigation was conducted with three replicates.

### Statistical analysis

All graphs’ statistical analysis and plotting were carried out using GraphPad Prism 7.0 (GraphPad Software Inc., San Diego, CA). ***p < 0.0001, **p < 0.01, and *p < 0.05 were considered as significant.

## Results

### Marine-bioinspired synthesis of Lam-AuNPs

**Fig. 1:**
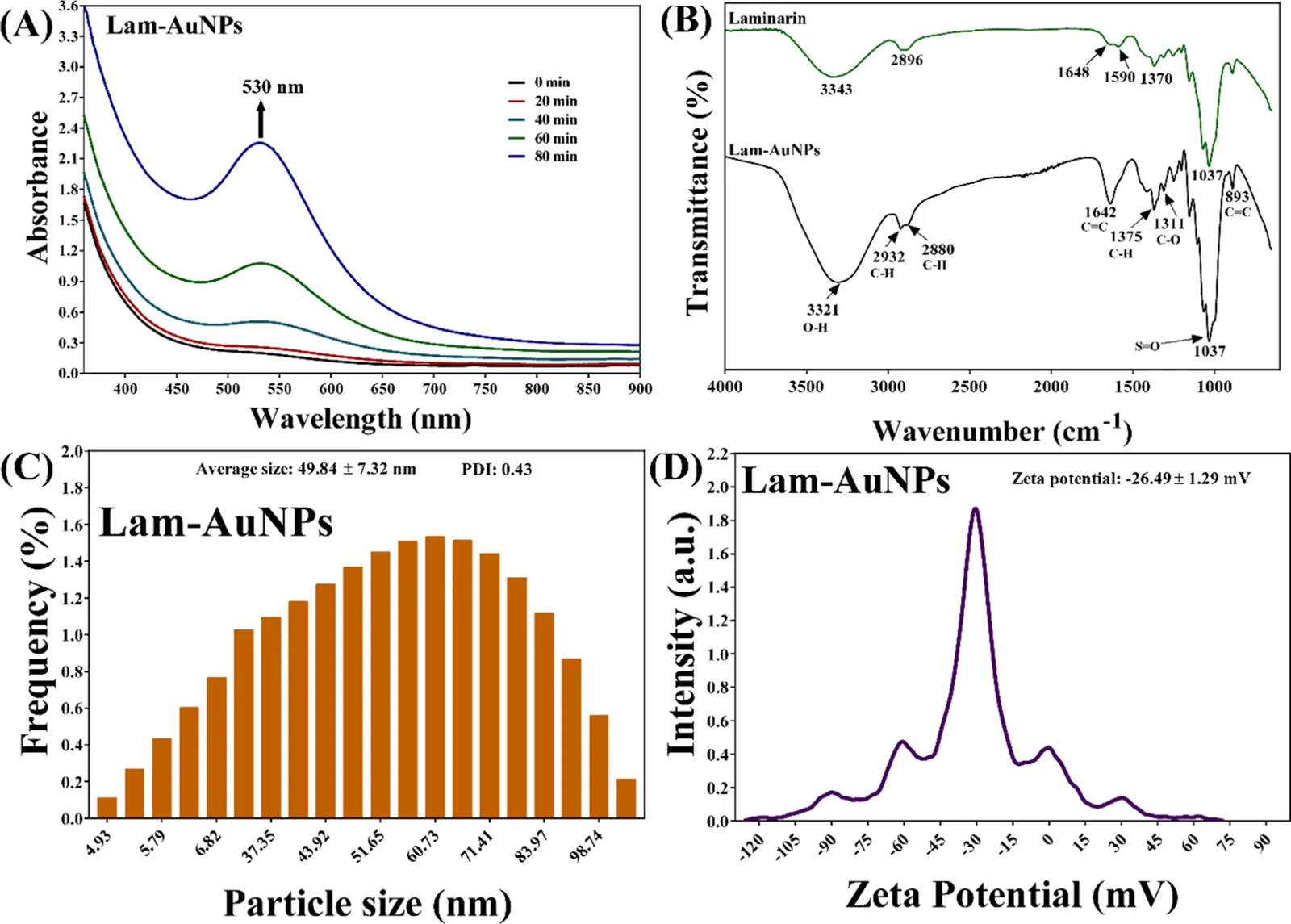
**A** In situ monitoring of the absorption spectra of Lam-AuNPs, **B** FTIR spectra, **C** size distribution, and **D** zeta potential

In this work, a marine-derived compound, such as Lam, was used for the synthesis of AuNPs. A comprehensive description of the steps involved in producing Lam-AuNPs is provided in the supplemental material (Fig. S1). The production of Lam-AuNPs is indicated by the deep-red-wine hue of the reaction containing the mixture of gold solution and Lam. Furthermore, UV–vis absorption spectra scanning revealed increased absorption spectra at 530 nm during the course of the reaction, which conforms to the production of the Lam-AuNPs (Fig. 1A). The FTIR study of Lam reveals 3343 cm^−1^, 2896 cm^−1^, 1648 cm^−1^, 1590 cm^−1^, 1370 cm^−1^, and 1037 cm^−1^. The peak at 3343 cm^−1^ corresponds to the O–H group, and the peak at 1037 cm^−1^ to the S = O sulfoxide group (Fig. 1B). The peak at 2896 cm^−1^ corresponds to the C-H starching of an alkane. The FTIR investigation revealed some distinctive spectra in Lam-AuNPs (Fig. 1B).

**Fig. 2:**
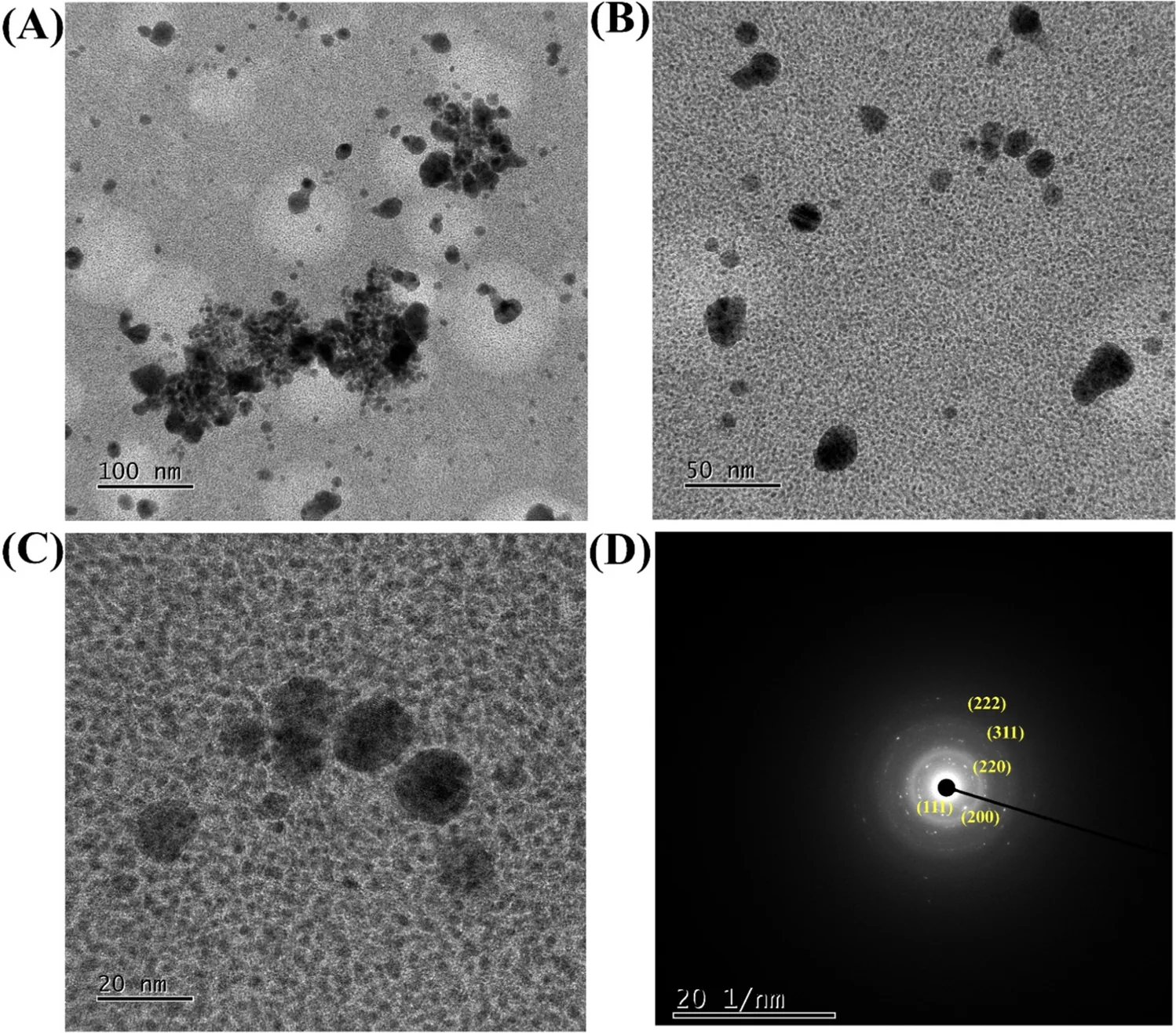
**A** FE-TEM micrograph at a resolution of 100 nm, **B** FE-TEM micrograph at a resolution of 50 nm, **C** FE-TEM micrograph at a resolution of 20 nm, and **D** SAED of Lam-AuNPs

The average size measurement by DLS analysis yields a value of 49.84 ± 7.32 nm (Fig. 1C). Furthermore, the polydispersity index value of 0.43 suggests that the Lam-AuNPs are homogeneous. The zeta potential of Lam-AuNPs was calculated to be − 26.49 ± 1.29 mV (Fig. 1D). The morphology of Lam-AuNPs imaged by FE-TEM was mostly spherical, although some irregular forms of NPs were also identified (Fig. 2). Figure 2A, B, and C show pictures of Lam-AuNPs at various resolutions.

**Fig. 3:**
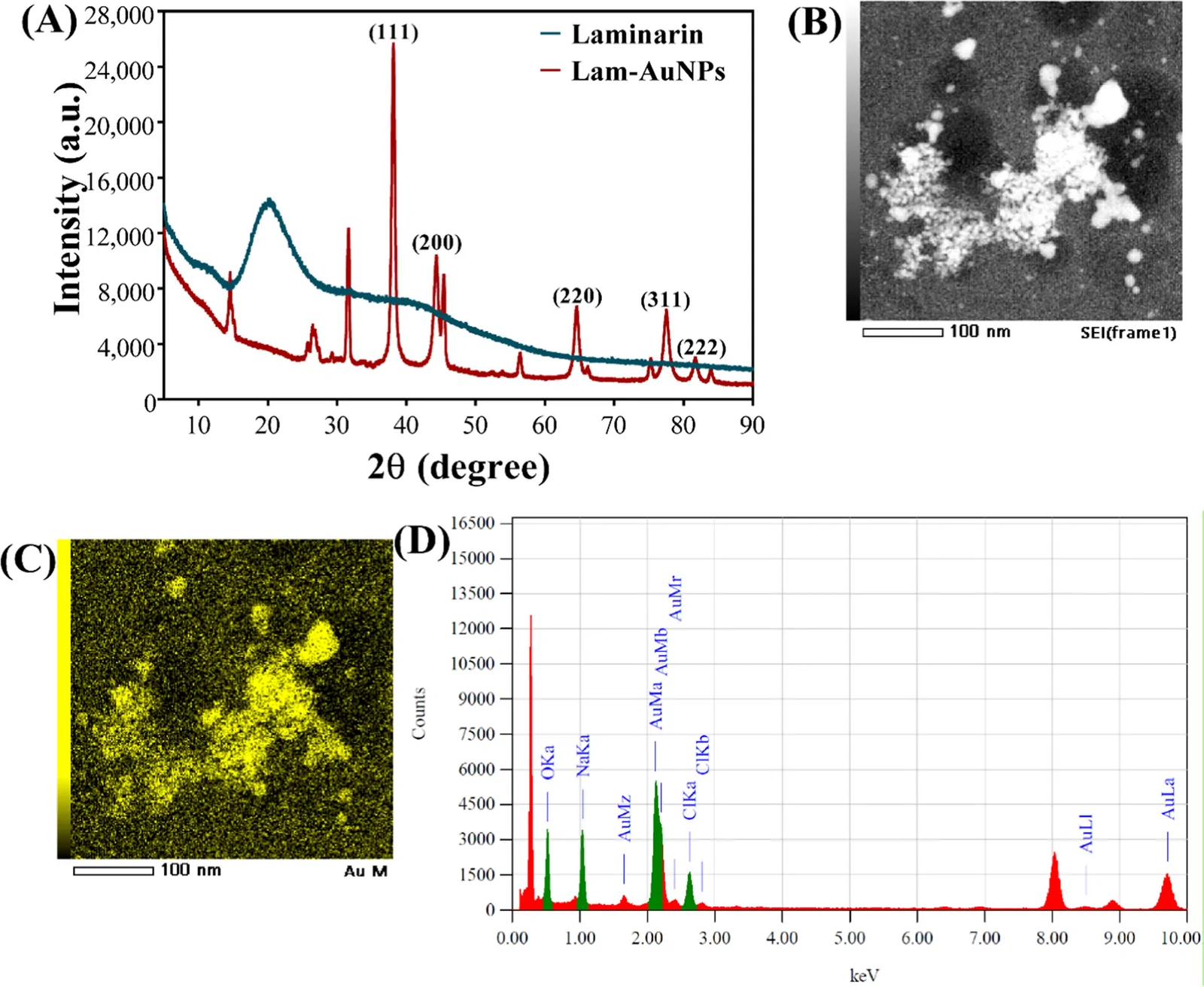
**A** Energy-dispersive XRD spectrum, **B** SEM image of Lam-AuNPs, **C** mapping of Au element, and **D** EDS spectra of Lam-AuNPs

In addition, the characterization of Lam-AuNPs involves an XRD analysis, which is then followed by elemental composition mapping (Fig. 3). The XRD analysis of pure Lam displays a broad peak in the range of 15 to 30° (Fig. 3A). On the other hand, the XRD analysis of Lam-AuNPs reveals a multitude of distinct peaks at different *2ϴ*, which correspond to 38.2°, 44.4°, 64.6°, 77.6°, and 81.7°, respectively (Fig. 3A). The (111), (200), (220), (311), and (222) planes of the face-centered cubic crystal structure are the ones that correspond to these peaks. As a result of the existence of these peaks in the XRD spectrum, the crystalline nature of the Lam-AuNPs has been confirmed. The crystalline nature of the Lam-AuNPs is shown by the bright circular fringes that can be seen in the SAED at the (111), (200), (220), (311), and (222) levels (Fig. 2D). The elemental mapping by EDS analysis reveals the presence of Au metal in the Lam-AuNPs (Fig. 3D). The spherical form of the Lam-AuNPs is also seen in the SEM picture in the EDS study. Based on several analytical instruments, the synthesis of Lam-AuNPs is confirmed.

### Antimicrobial effect of Lam-AuNPs

The MIC values of Lam-AuNPs against many drug-resistant microbiological pathogens investigated in standard and host-mimicking media are provided in the supplemental material (Table S1). The results demonstrated that the MIC value of Lam-AuNPs in a TSB medium is 256 μg/mL against *P. aeruginosa* and *S. aureus*. When evaluated in an artificial sputum medium, a higher MIC value (512 μg/mL) was found. However, in SHU, the MIC values of Lam-AuNPs against *P. aeruginosa* and *S. aureus* were reported to be 128 μg/mL, which is onefold lower than the MIC value measured in the TSB medium. Surprisingly, the MIC value of Lam-AuNPs in artificial saliva was found to be 4 μg/mL, which is fivefold and sixfold lower than the MIC value measured in TSB and artificial sputum medium, respectively.

In the TSB, the MIC value for *E. coli* was determined to be 256 μg/mL. This value is comparable to the MIC value against *P. aeruginosa* and *S. aureus*. The MIC value against *E. coli* was 128 μg/mL in the sputum, 32 μg/mL in the SHU, and 8 μg/mL in the saliva. The MIC value against *K. pneumoniae* in the TSB was more than 1024 µg/mL, while 512 µg/mL in the sputum. The MIC values for *K. pneumoniae* in the SHU and saliva were 16 µg/mL and 4 µg/mL, respectively. The MIC value against *L. monocytogenes* in the TSB was 512 µg/mL, but it is greater in the case of SHU (> 512 µg/mL). The MIC value against *L. monocytogenes* in sputum and saliva was determined to be 256 µg/mL and > 2.0 µg/mL, respectively. The MIC value against *S. mutans* in the TSB was 512 µg/mL, which is 128 µg/mL in the saliva. The MIC value against *S. mutans* in urine and sputum could not be determined. The MIC value against *C. albicans* in PDB and saliva was discovered to be 1024 µg/mL and 512 µg/mL, but the MIC value in sputum and SHU was determined to be 128 µg/mL. According to the findings, the MIC values in the artificial saliva and SHU medium were lowered against all investigated microbiological pathogens.

### Biofilm inhibitory effect of Lam-AuNPs

**Fig. 4:**
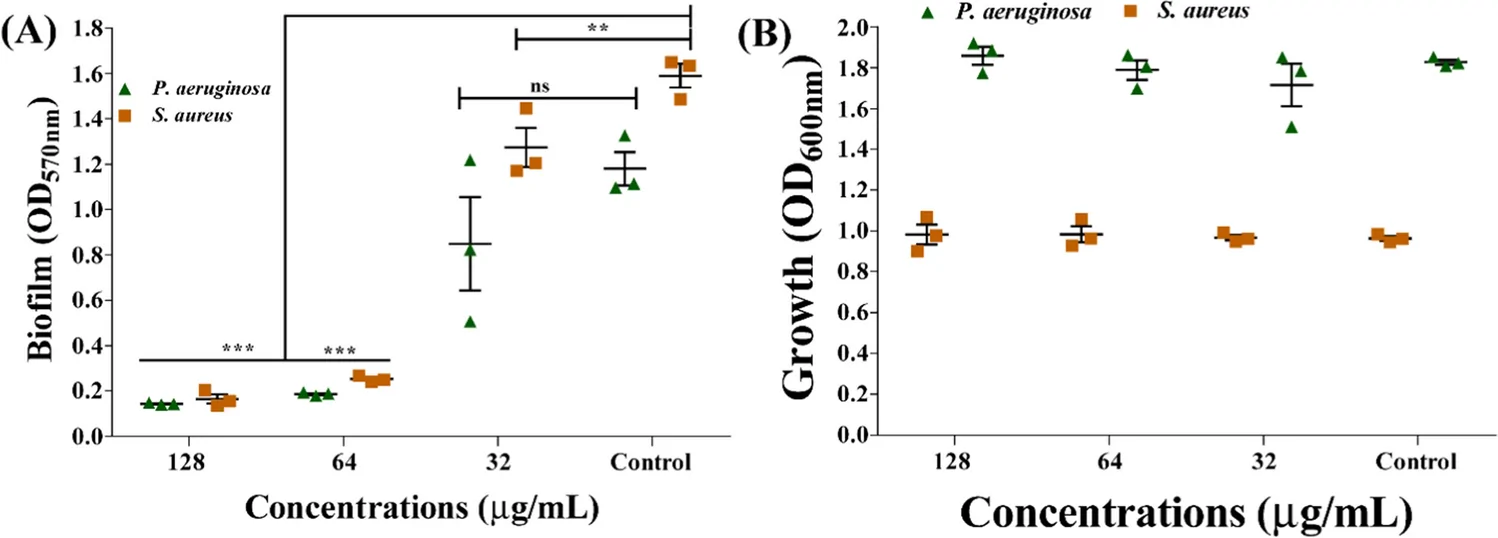
Biofilm inhibitory effects of Lam-AuNPs towards bacterial pathogens. **A** Biofilm inhibition of *P. aeruginosa* and *S. aureus* and **B** growth properties of *P. aeruginosa* and *S. aureus*. ***p < 0.0001 and **p < 0.01 were considered significant

**Fig. 5:**
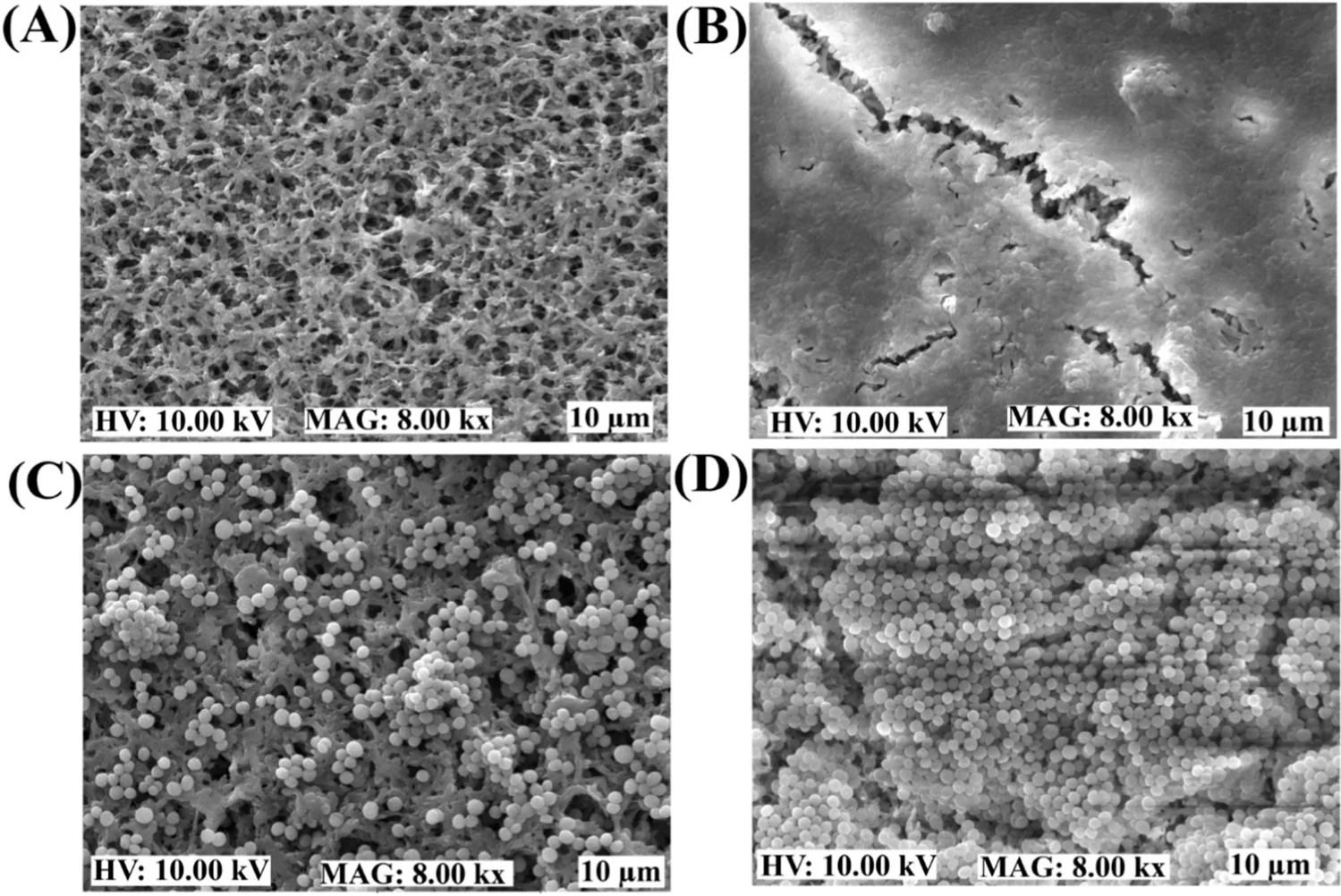
Microscopic imaging of the biofilm cells in the presence of Lam-AuNPs. **A** *P. aeruginosa* biofilm treated with Lam-AuNPs, **B** *P. aeruginosa* biofilm control, **C** *S. aureus* biofilm treated with Lam-AuNPs, and **D** *S. aureus* biofilm control

The sub-MIC was used to evaluate the efficiency of Lam-AuNPs as antibiofilm agents against *P. aeruginosa* and *S. aureus* in TSB and the host-mimicking medium. The biofilm inhibitory effects on *P. aeruginosa* and *S. aureus* were discovered to be dose-dependent (Fig. 4). Maximum inhibition was reported to be 87.89% and 89.65% against *P. aeruginosa* and *S. aureus*, respectively, at a concentration of 128 μg/mL of Lam-AuNPs (Fig. 4A). The sub-MIC levels of Lam-AuNPs tested did not limit growth (Fig. 4B), indicating that the cells are free to form biofilms on the surface. The microscopic visualization of the biofilm cell in the presence of the Lam-AuNPs was also studied to confirm its antibiofilm action. The SEM analysis revealed that the Lam-AuNPs treatment inhibited the surface attachment of *P. aeruginosa* cells the most (Fig. 5A), compared to the control cells, which produced a thick biofilm (Fig. 5B). Similarly when treated with Lam-AuNPs, the *S. aureus* biofilm on the membrane surface was extremely few (Fig. 5C), but a dense biofilm of control cells was observed (Fig. 5D).

**Fig. 6:**
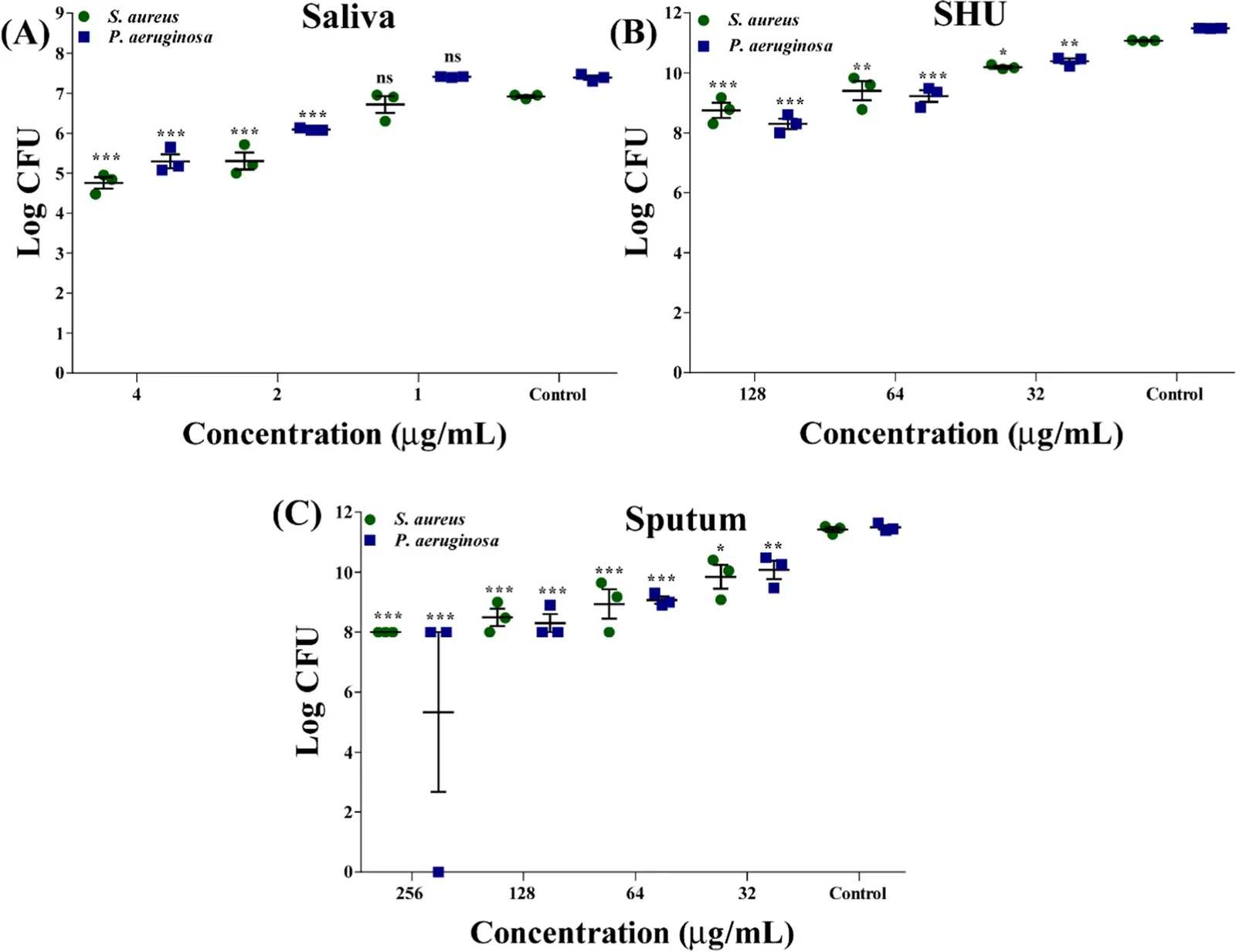
Biofilm inhibition effect of Lam-AuNPs in the different types of host-mimicking media. **A** Log CFU value of *P. aeruginosa* and *S. aureus* in the artificial human saliva media, **B** log CFU values of *P. aeruginosa* and *S. aureus* in SHU media, and **C** log CFU value of *P. aeruginosa* and *S. aureus* in the artificial sputum media. ***p < 0.0001, **p < 0.01, and *p < 0.05 were considered as significant

The sub-MIC value of Lam-AuNPs also exhibited the concentration-dependent inhibition of *P. aeruginosa* and *S. aureus* biofilms in different types of host-mimicking medium (Fig. 6). At a concentration of 4 μg/mL, Lam-AuNPs in artificial saliva, the log CFU value of *P. aeruginosa* and *S. aureus* cells was decreased to 2.09 and 2.16, respectively (Fig. 6A). The results indicate that *P. aeruginosa* and *S. aureus* biofilms were suppressed to 28.30% and 31.23%, respectively, in artificial saliva at 4 μg/mL concentrations. The inhibition of *P. aeruginosa* and *S. aureus* biofilms in SHU was greatest at 128 μg/mL concentrations. The reduction in log CFU value of *P. aeruginosa* and *S. aureus* cells at 128 μg/mL Lam-AuNPs concentration was 3.18 and 2.32, respectively (Fig. 6B). At a dose of 128 μg/mL, the percentage of biofilm inhibition of *P. aeruginosa* and *S. aureus* in the SHU was determined to be 27.73% and 20.96%, respectively. The biofilm inhibition of *P. aeruginosa* and *S. aureus* in the SHU was three times lower at the same dose (128 μg/mL) as in the TSB.

Similarly, at 256 μg/mL, the suppression of *P. aeruginosa* and *S. aureus* biofilm in artificial sputum media was shown to be significant (Fig. 6C). At 256 μg/mL concentration, the log CFU values of *P. aeruginosa* and *S. aureus* in sputum medium were 6.16 and 3.41, respectively. The percentages of inhibition of *P. aeruginosa* and *S. aureus* biofilms in sputum medium at 256 μg/mL were determined to be 53.59% and 29.93%, respectively. The percentage of biofilm inhibition against *P. aeruginosa* and *S. aureus* in sputum media at a concentration of 128 μg/mL was reported to be 27.77% and 25.62%, respectively, which is three times lower than the inhibition seen in the TSB medium. According to the findings, Lam-AuNPs can effectively inhibit the early stages of biofilm formation in *P. aeruginosa* and *S. aureus*. However, the inhibitory impact was observed to vary in standard and host-mimicking media.

### Dispersal of mature biofilm

**Fig. 7:**
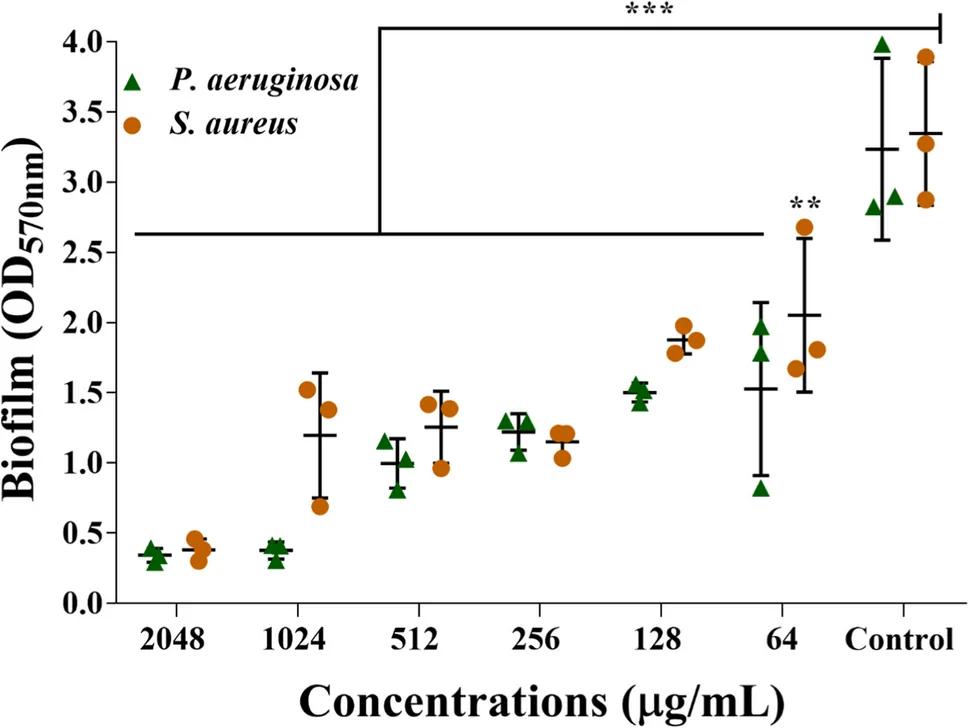
Eradication of mature biofilms of *P. aeruginosa* and *S. aureus* by Lam-AuNPs. ***p < 0.0001 and **p < 0.01 were considered as significant

The destruction of the fully mature biofilm of *P. aeruginosa* and *S. aureus* that had been established was also evaluated using Lam-AuNPs at concentrations that were at the MIC, above the MIC, and under the sub-MIC (Fig. 7). The eradication efficiency of *P. aeruginosa* mature biofilm was reported to be 62.26% at the MIC value (256 μg/mL) of Lam-AuNPs. However, the eradication efficiency was enhanced by 88.35% and 89.42% at the higher MIC values (1024 μg/mL and 2048 μg/mL) of Lam-AuNPs. Similarly, the eradication of *S. aureus* mature biofilm was reported to be 65.62% at the MIC level (256 μg/mL) but increased to 64.25% and 88.62% when higher MIC values (1024 and 2048 μg/mL) of Lam-AuNPs were utilized. The findings support the notion that the mature biofilm of these bacterial pathogens is very resistant to antimicrobial agents.

### Virulence attenuating properties of Lam-AuNPs

**Fig. 8:**
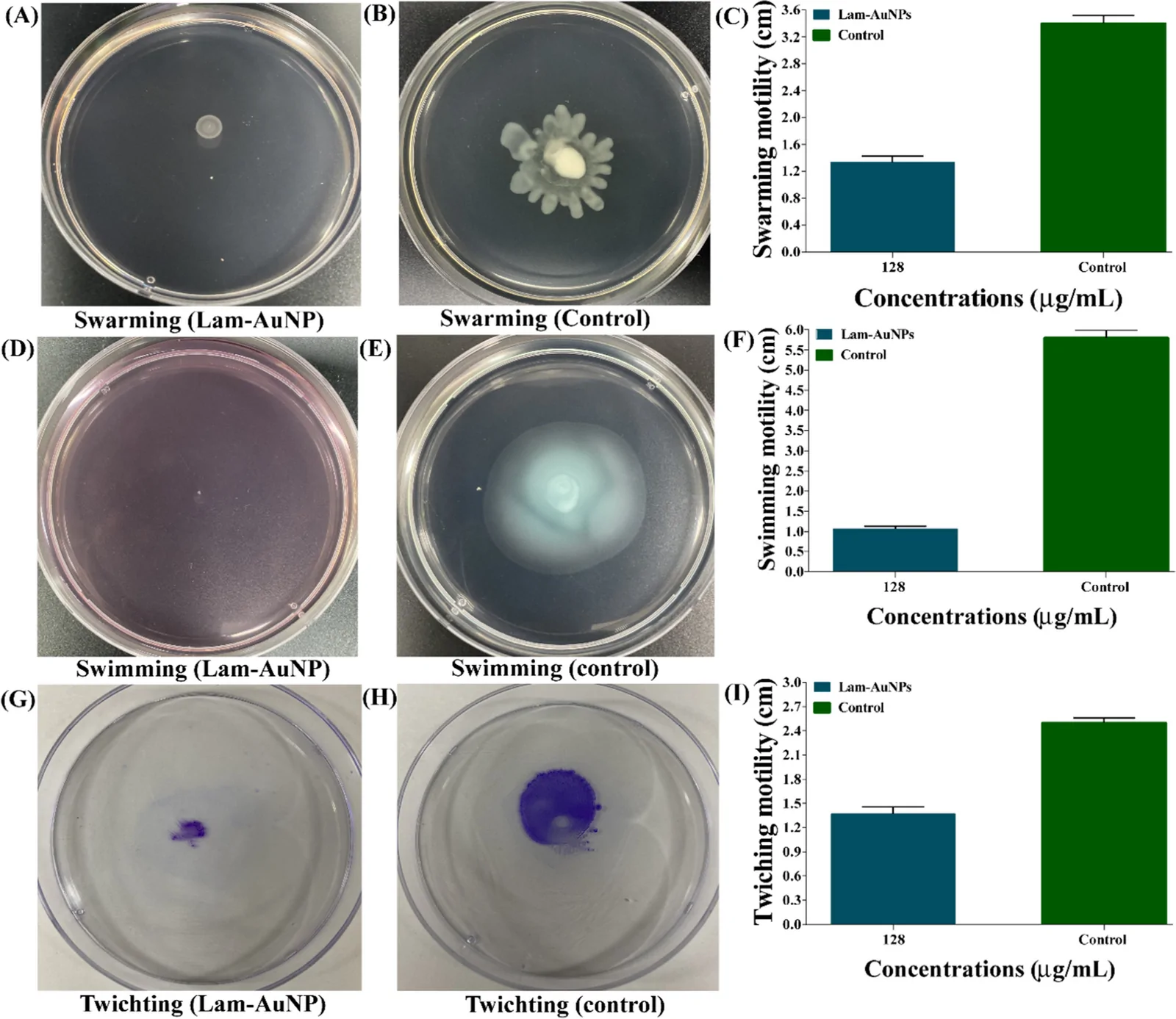
Inhibition of different types of motility properties of *P. aeruginosa* by Lam-AuNPs. **A** Swarming treated with Lam-AuNPs, **B** swarming control, **C** bar graph of swarming, **D** swimming treated with Lam-AuNPs, **E** swimming control, **F** bar graph of swimming, **G** twitching treated with Lam-AuNPs, **H** twitching control, and **I** bar graph of twitching

The sub-MIC inhibitory effect of Lam-AuNPs on different types of virulence traits in *P. aeruginosa* and *S. aureus* was investigated. The Lam-AuNPs had a significant influence on the swarming, swimming, and twitching motility of *P. aeruginosa* when they were present at concentrations of 128 μg/mL (Fig. 8). Figure 8A and B show a typical picture of an agar plate displaying swarming movement of *P. aeruginosa* cells. The highest swarming motility inhibition was reported to be 60.8% (Fig. 8C). Figure 8D and E show the agar plate images of swimming motility in which Lam-AuNPs are present and absent (control). The highest inhibition of swimming motility was 81.6% (Fig. 8F). The pili-mediated twitching motility was maximally suppressed at 45.3% (Fig. 8I), as evidenced on the agar plate (Fig. 8G and H).

**Fig. 9:**
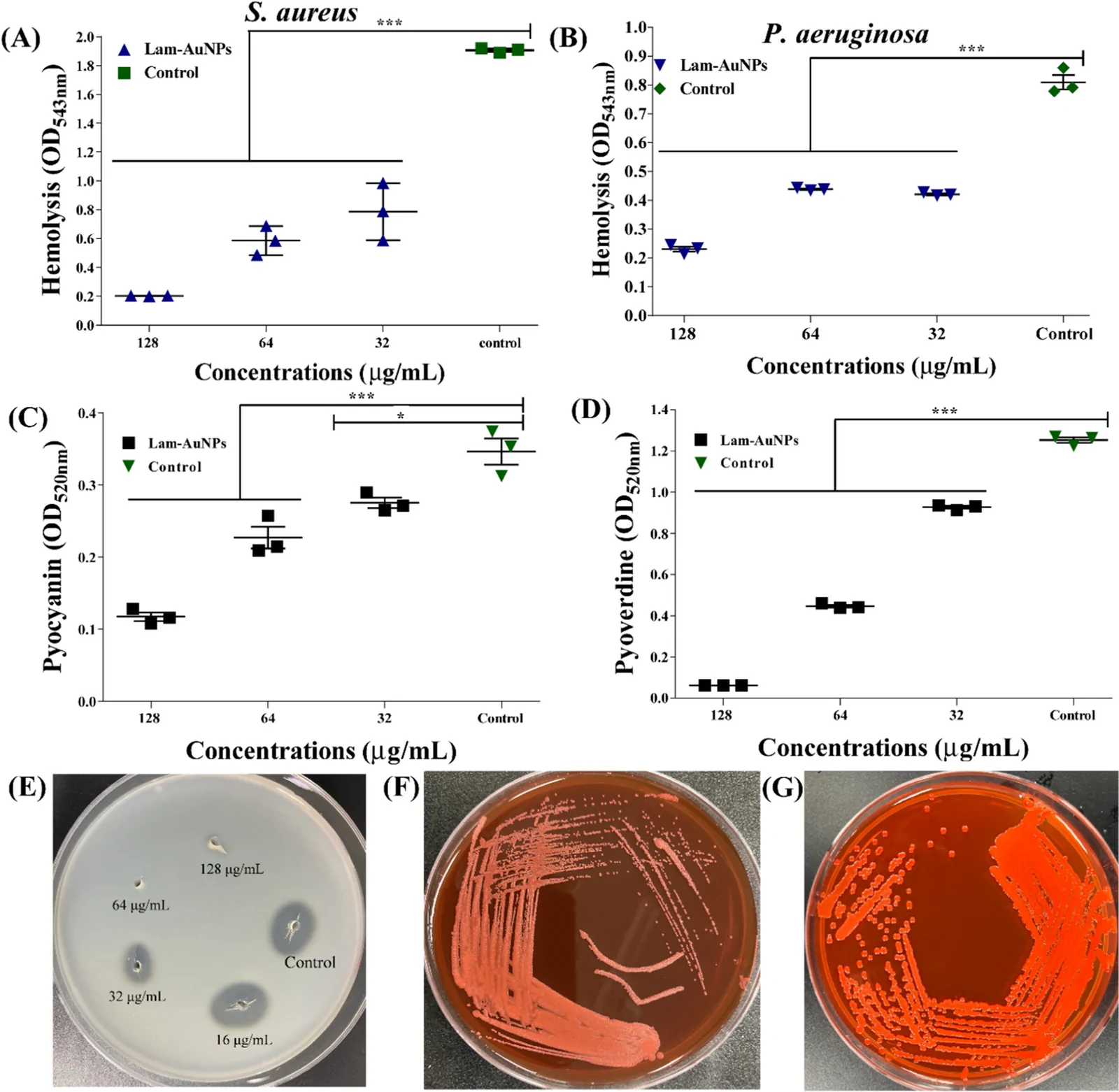
Inhibition of multiple virulence properties of *P. aeruginosa* and *S. aureus* by Lam-AuNPs. **A** Hemolysis of *S. aureus*, **B** hemolysis of *P. aeruginosa*, **C** pyocyanin production from *P. aeruginosa*, **D** pyoverdine production from *P. aeruginosa*, **E** protease activity of *P. aeruginosa*, **F** Congo-red staining of *S. aureus* treated with Lam-AuNPs, and **G** Congo-red staining of *S. aureus* control. ***p < 0.0001, **p < 0.01, and *p < 0.05 were considered as significant

Lam-AuNPs considerably decreased the hemolytic activity of *P. aeruginosa* and *S. aureus* (Fig. 9A and B). The hemolytic activity of *P. aeruginosa* and *S. aureus* was decreased to 71.49% and 89.38%, respectively, at 128 μg/mL (Fig. 9A and B). In addition, Lam-AuNPs were examined to see whether they can inhibit the virulence characteristics of *P. aeruginosa*, including pyocyanin, pyoverdine, and protease activity. The inhibition of pyocyanin and pyoverdine was concentration-dependent (Fig. 9C and D). The maximal inhibition of pyocyanin and pyoverdine by Lam-AuNPs at 128 μg/mL was determined to be 66.15% and 95.07%, respectively. Similarly, suppression of protease activity on the skim-milk agar plate was concentration-dependent (Fig. 9E). The Congo-red staining technique was used to assess amyloid fibril formation, which is another virulence component in *S. aureus*. The colony color of *S. aureus* was observed to be somewhat lighter in the presence of Lam-AuNPs compared to the control, which had a red, rough, and dry colony (Fig. 9F and G). Based on the findings, Lam-AuNPs can inhibit or suppress various virulence features of *P. aeruginosa* and *S. aureus*.

### Cell cytotoxicity assay of Lam-AuNPs

**Fig. 10:**
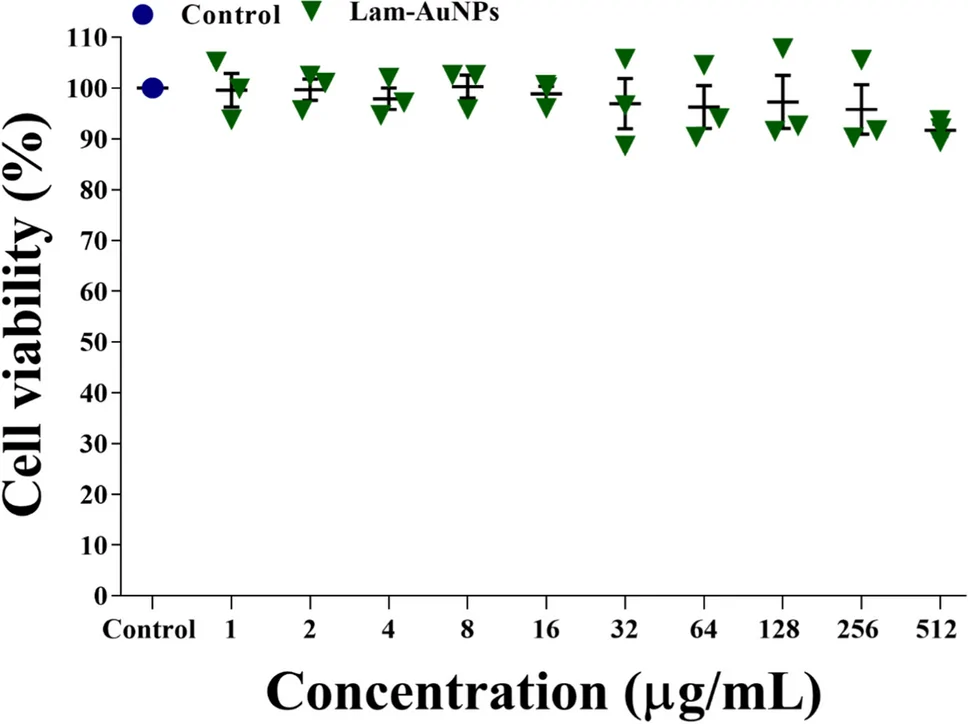
Cell cytotoxicity effects of Lam-AuNPs towards mouse macrophase RAW 264.7 cell lines

The cell toxicity effects of Lam-AuNPs on animal cell cultures like mouse macrophage RAW 264.7 were investigated. There is no cytotoxicity at concentrations ranging from 1 to 512 μg/mL (Fig. 10). The cytotoxicity impact was detected when Lam-AuNP concentrations were raised over 512 μg/mL. The concentration of Lam-AuNPs employed for antibiofilm, antibacterial, and antivirulence activities was discovered to be independent of the concentration at which cytotoxicity was detected.

## Discussion

The failure of antimicrobial therapy to bacterial infection due to the development of AMR leads to the discovery of various alternative treatments (Murray et al. 2022; Ventola 2015). In the current investigation, we chose *P. aeruginosa* and *S. aureus* as biofilm-forming bacterium pathogens against which to develop drugs. The approach adopted the use of NPs to control biofilm-forming bacterial pathogens because of the high success and several benefits of using NPs to control microbial infection (Jeong et al. 2022; Kang et al. 2023; Khan et al. 2022, 2021). Furthermore, to apply green-chemistry-based NP synthesis, a marine-derived polymeric material, Lam, was used in the synthesis of the metal NPs. The AuNPs were synthesized utilizing Lam as a reducing agent in conditions mentioned in different types of publications (Remya et al. 2018; Yugay et al. 2020). Several advanced instruments were employed to fully characterize the Lam-AuNPs produced. The initial in situ confirmation of the Lam-AuNPs was verified by the emergence of the red-wine color of the solution mixture during the time reaction. According to many studies, forming the wine-red shade of the mixture solution is the first stage in confirming AuNPs (Kang et al. 2023; Khan et al. 2023; Tabassum et al. 2023b). Furthermore, the UV–visible absorption spectra observed at 530 nm during the in situ synthesis of the Lam-AuNPs support its production. The absorption spectrum of Lam-AuNPs is quite close to that of previously produced AuNPs from various natural sources (El-Deeb et al. 2022; Kaithavelikkakath Francis et al. 2020). Furthermore, some distinctive FTIR spectra suggest the contribution of a specific group in the production of AuNPs. These FTIR peaks have previously been detected in green-synthesized AuNPs (Khan et al. 2019). The size of the NPs is a key characteristic for successful action against microbial pathogens (Menichetti et al. 2023). The size of the Lam-AuNPs is determined to be 49.84 ± 7.32 nm, which is within the needed size range for efficient antibacterial activity. The AuNPs produced with marine-derived phloroglucinol had a comparable size (41.6 ± 3.9 nm) to the Lam-AuNPs (Khan et al. 2021). Similarly, marine-derived polymer fucoidan-based synthetic AuNPs were discovered to have comparable sizes (Kang et al. 2022; Khan et al. 2019). The morphology of AuNPs was observed to vary depending on the type of agents used (Ibrahim et al. 2019; Piktel et al. 2021). The morphology of the Lam-AuNPs was discovered to be spherical, which is consistent with the spherical form of AuNPs produced utilizing other natural products (Kang et al. 2022; Khan et al. 2019). The antibacterial activity of the synthesized Lam-AuNPs against *P. aeruginosa* and *S. aureus* was tested in several synthetic media, such as standard and host-mimicking. The MIC values of Lam-AuNPs against *P. aeruginosa* and *S. aureus* varied according to medium (Table S1). The MIC value in TSB was determined to be 256 μg/mL, but the MIC value in the sputum medium (512 μg/mL) was onefold greater. Surprisingly, the MIC value of Lam-AuNPs in SHU and artificial saliva was onefold and fivefold lower compared to the MIC value in TSB. Additionally, the MIC value of Lam-AuNPs against several other drug-resistant microbial pathogens in standard and artificial host-mimicking media varied depending on the media type, with some values in the same order as determined against *S. aureus* and *P. aeruginosa* (Table S1). Previous research indicated that the MIC value of Lam-AuNPs against fish pathogens such as *Aeromonas hydrophila* was 2.25 μg/mL, which is 113 times lower than the MIC value of Lam-AuNPs against *S. aureus* and *P. aeruginosa* in standard growth media (Vijayakumar et al. 2021).

The higher MIC value in sputum media than in normal media is consistent with the previously determined MIC value for colistin drugs against *P. aeruginosa* (Sweeney et al. 2020). The reduced MIC values in the host-mimicking media of artificial saliva and SHU suggest that certain compounds in these media may have a synergistic/additive activity or serve as adjuvants for the action (Ersoy et al. 2017; Tabassum et al. 2023a). It has been discovered that artificial saliva contains a histatin peptide, which possesses antibacterial and antifungal properties (Du et al. 2017; Sharma et al. 2021). Recent research also found that the MIC value of phloroglucinol-AuNPs against *S. aureus* and *C. albicans* in artificial saliva is lower than the MIC value in standard media (Tabassum et al. 2023a). When employed in the in vivo system, determining the MIC values in the host-mimicking medium may replicate the host environment (Crabbé et al. 2014). Previous studies revealed that the MIC value of fucoidan-AuNPs in TSB medium against *P. aeruginosa* was 512 μg/mL, which was onefold higher than Lam-AuNPs (Khan et al. 2019).

After determining the MIC concentrations, the Lam-AuNPs were tested for their effects on biofilm formation and virulence qualities against *P. aeruginosa* and *S. aureus*. Since biofilm has become one of the principal resistance mechanisms of most biofilm-forming bacterial infections, it has also become one of the physical key barriers to the entrance of antimicrobial agents (Mah and O’Toole 2001). The sub-MIC of Lam-AuNPs inhibited early-stage biofilm formation in the TSB and the host-mimicking media. At 128 μg/mL, the maximal inhibition of *P. aeruginosa* and *S. aureus* biofilm was reported to be 87.89% and 89.65%, respectively.

*P. aeruginosa* and *S. aureus* biofilm inhibition by Lam-AuNPs is concentration-dependent. Earlier studies have shown that Lam-AuNPs, when present at a dosage of 100 µg/mL, demonstrate a noteworthy suppression of the initial-stage biofilm of the fish pathogen *A. hydrophila* (Vijayakumar et al. 2021). Moreover, the biofilm inhibition against *A. hydrophila* was shown to be concentration-dependent. When examined in different host-mimicking conditions, there is a variance in maximal biofilm inhibition (Fig. 6). The variation in Lam-AuNP concentrations in the TSB and host-mimicking media to obtain maximal biofilm inhibition has highlighted the importance of not ignoring the host-mimicking media when assessing the antibiofilm activity of any agents (Sweeney et al. 2020). Based on the observation that the disruption of mature *P. aeruginosa* and *S. aureus* biofilms matrix occurred at greater concentrations of Lam-AuNPs (MIC and above MIC values), it can be concluded that the biofilm complex acts as a substantial barrier to the entrance of drugs (Stewart 2002). Previously, spontaneously generated AuNPs eliminated the formed mature biofilm of *P. aeruginosa* and *S. aureus* at MIC and beyond MIC levels (Kang et al. 2023, 2022; Khan et al. 2021, 2019). Motility, protease, siderophore, hemolytic activity, pyocyanin, rhamnolipid, and amyloid fibril are all known to play essential roles in host cell attachment and invasion (Cheung et al. 2021; Jeong et al. 2023; Liao et al. 2022). As a result, disarming *P. aeruginosa* and *S. aureus* virulence features has been identified as another possible way to reduce pathogen infection (Khan et al. 2019; Zhu et al. 2015). Because the motility of *P. aeruginosa*, which includes characteristics such as swarming, swimming, and twitching, leads to more biofilm advancement and the process of surface adhesion (O’Toole and Kolter 1998), inhibiting motility using Lam-AuNPs might be an option for controlling biofilm and infections. At sub-MIC levels, Lam-AuNPs effectively suppress swarming, swimming, and twitching motilities, consistent with the inhibitory action of AuNPs produced earlier utilizing other natural products (Kang et al. 2023, 2022; Khan et al. 2021, 2019). It was discovered that other virulence characteristics of *P. aeruginosa*, including hemolysis, pyocyanin, pyoverdine, and protease activity, were suppressed in a way that was dependent on the concentration of the Lam-AuNPs. Similar antivirulence effects of AuNPs synthesized from phloroglucinol, fucoidan, *Leuconostoc* sp. strain C2, and *Lactiplantibacillus* sp. strain C1 also have been reported previously in *P. aeruginosa* (Kang et al. 2023, 2022; Khan et al. 2021, 2019). The presence of Lam-AuNPs greatly decreased virulence features in *S. aureus*, such as hemolysis and amyloid fibril formation, indicating potential antivirulence agents. When NPs with potential biological activity are biocompatible, they can be applied (Kyriakides et al. 2021). The biocompatibility of Lam-AuNPs was also tested using macrophage cell lines, and the findings revealed that there was no cytotoxicity at the concentrations utilized for antibiofilm and antivirulence properties (Fig. 10). At a dose of 100 µg/mL, the Vero cell line, which was derived from the kidney of an African green monkey, did not exhibit any cytotoxic effects when treated with Lam-AuNPs, as shown by previous findings (Vijayakumar et al. 2021). The present findings are also consistent with the non-cytotoxicity of antibacterial concentrations of phloroglucinol-AuNPs in the macrophage cell line (Khan et al. 2021).

In conclusion, this work aimed to synthesize the Lam-AuNPs using a marine-derived polymer, Lam, which is then completely characterized using different experimental techniques. The Lam-AuNPs display antibacterial action against *P. aeruginosa* and *S. aureus* in normal and host-mimicking conditions. When compared to the TSB and sputum medium, the MIC values in artificial saliva and SHU were lower. The first-stage biofilm of *P. aeruginosa* and *S. aureus* is significantly inhibited by Lam-AuNPs at the sub-MIC level. In addition, the MIC and above MIC value of Lam-AuNPs can remove the mature biofilm typically created by these bacteria. It has also been shown that the sub-MIC level of Lam-AuNPs inhibits the formation of pyocyanin and pyoverdine in *P. aeruginosa*, as well as the motility, hemolytic activity, and protease activity. Lam-AuNPs greatly suppressed the development of amyloid fibril virulence factors and hemolysis in *S. aureus*. The Lam-AuNPs produced had no cytotoxicity impact on the animal cell line, indicating their biocompatibility. Based on the antibacterial activity demonstrated in host-mimicking conditions and biocompatibility, the synthesized Lam-AuNPs can be used as an effective agent in the healthcare system to control the biofilm and virulence features of *P. aruginsoa* and *S. aureus*. Future research should be conducted to determine how Lam-AuNPs suppress the initial-stage biofilm and reduce numerous virulence features in bacterial pathogens by gene expression analysis of relevant genes.

## Supplementary Information

Below is the link to the electronic supplementary material.

Supplementary file1 (PDF 118 KB)

## Data Availability

Not applicable.
