# Supplementary material for: Antibiofilm and antivirulence activities of laminarin-gold nanoparticles in standard and host-mimicking media
Source: Appl Microbiol Biotechnol. 2024 Feb 13;108:203. doi: 10.1007/s00253-024-13050-4 (PMC10864539; doi:10.1007/s00253-024-13050-4)
Supplement: Supplementary file 1 [file 253_2024_13050_MOESM1_ESM.pdf]

Supplementary Information:

Title: Antibiofilm and antivirulence activities of laminarin-gold nanoparticles in standard and host-mimicking media

Authors: Nazia Tabassum[1,2], Fazlurrahman Khan[1,2,3*] Geum-Jae Jeong[4], Dokyung Oh[1,2,4] and Young-Mog Kim[1,2,4*]

[1]*Marine Integrated Biomedical Technology Center, The National Key Research Institutes in Universities, Pukyong National University, Busan 48513, Republic of Korea.*

[2]*Research Center for Marine Integrated Bionics Technology, Pukyong National University, Busan 48513, Republic of Korea*

[3]*Institute of Fisheries Sciences, Pukyong National University, Busan 48513, Republic of Korea*

[4]*Department of Food Science and Technology, Pukyong National University, Busan 48513, Republic of Korea*

*Corresponding authors: Fazlurrahman Khan and Young-Mog Kim

Phone: +82-51-629-5832; Fax: +82-51-629-5824

E-mail: fkhan055@pknu.ac.kr and ymkim@pknu.ac.kr

Running title: Antibiofilm and antivirulence properties of Lam-AuNPs

Table S1. The MIC values of Lam-AuNPs in complex and various types of hots-mimicking media against various pathogenic bacteria, which are multi-drug resistance

**Synthetic media**	**MIC (µg/mL)**						
	**PA**	**SA**	**SM**	**CA**	**EC**	**KP**	**LM**
TSB/PDB	256	256	512	1024	256	1024	512
Artificial sputum media	512	512	ND	128	128	512	256
Synthetic human urine	128	128	ND	128	32	16	> 512
Artificial saliva	4	4	128	512	8	4	> 2.0

ND, Note determined; TSB, tryptic soya broth; PDB, potato dextrose broth; PA, *Pseudomonas aeruginosa*; SA, *Staphylococcus aureus*; SM, *Streptococcus mutans*; CA, *Candida albicans*; EC, *Escherichia coli*; KP, *Klebsiella pneumoniae*; and LM, *Listeria monocytogenes*.

The MIC value was determined by the micro broth dilution method by incubating the cell culture. The MIC value in the standard growth media was decided based on the OD_{600} values. However, the colony-counting method was used in the host-mimicking media to determine the MIC values (Tabassum et al. 2023).

Fig. S1. Steps involved in the synthesis of Lam-AuNPs and its instrumental characterization

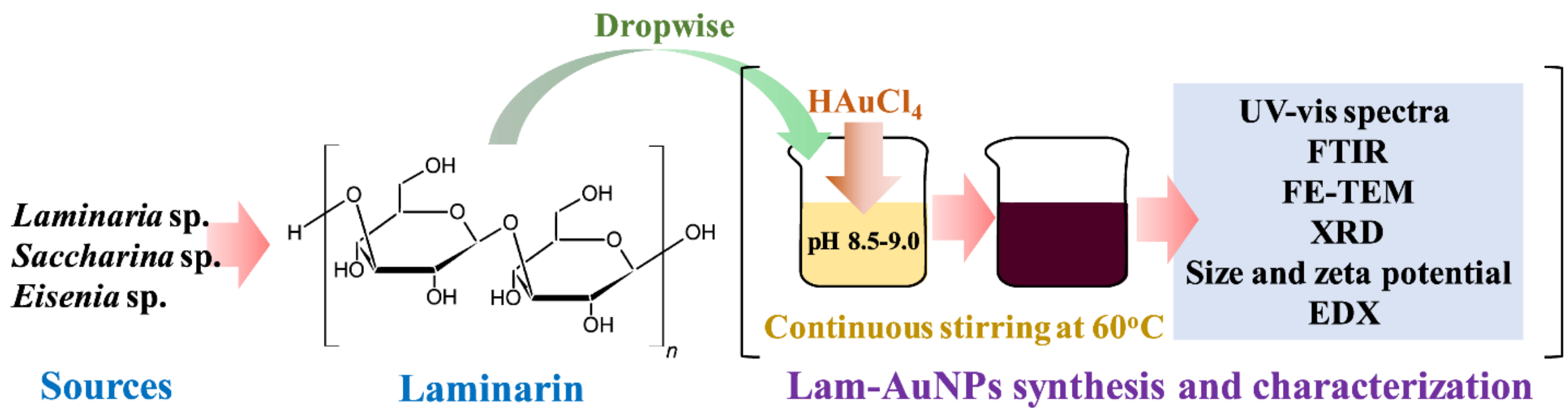

References

Tabassum N, Jeong G-J, Jo D-M, Khan F, Kim Y-M (2023a) Treatment of *Staphylococcus aureus* and *Candida albicans* polymicrobial biofilms by phloroglucinol-gold nanoparticles. Microb Pathog 185:106416 doi: 10.1016/j.micpath.2023.106416
